# Design and Implementation of a Cascade Control System for a Variable Air Volume in Operating Rooms Based on Pressure and Temperature Feedback

**DOI:** 10.3390/s25185656

**Published:** 2025-09-10

**Authors:** Abdulmohaymin Bassim Qassim, Shaimaa Mudhafar Hashim, Wajdi Sadik Aboud

**Affiliations:** 1 Department of Artificial Intelligence & Robotics Engineering, Al-Nahrain University, P.O. Box 64040, Al Jadriyah, Baghdad 10072, Iraq; shaimaa.mudhafar@nahrainuniv.edu.iq (S.M.H.); wajdi.sadiq@nahrainuniv.edu.iq (W.S.A.); 2 HEXACORP-AHI Carrier FZC (Automated Logic Corporation—A Carrier Company), Karrada—Al-Hurriya sq. 925/25/62-1, Baghdad 10069, Iraq

**Keywords:** VAV air-conditioning systems, building automation system, temperature and pressure control of operating rooms, automated logic corporation, cascade control system, direct digital controller

## Abstract

This research presents the design and implementation of a cascade Proportional–Integral (PI) controller tailored for a Variable Air Volume (VAV) system that was specially created and executed particularly for hospital operating rooms. The main goal of this work is to make sure that the temperature and positive pressure stay within the limits set by ASHRAE Standard 170-2017. This is necessary for patient safety, surgical accuracy, and system reliability. The proposed cascade design uses dual-loop PI controllers: one loop controls the temperature based on user-defined setpoints by local control touch screen, and the other loop accurately modulates the differential pressure to keep the pressure of the environment sterile (positive pressure). The system works perfectly with Building Automation System (BAS) parts from Automated Logic Corporation (ALC) brand, like Direct Digital Controllers (DDC) and Web-CTRL software with Variable Frequency Drives (VFDs), advanced sensors, and actuators that give real-time feedback, precise control, and energy efficiency. The system’s exceptional responsiveness, extraordinary stability, and resilient flexibility were proven through empirical validation at the Korean Iraqi Critical Care Hospital in Baghdad under a variety of operating circumstances. Even during rapid load changes and door openings, the control system successfully maintained the temperature between 18 and 22 °C and the differential pressure between 3 and 15 Pascals. Four performance scenarios, such as normal (pressure and temperature), high-temperature, high-pressure, and low-pressure cases, were tested. The results showed that the cascade PI control strategy is a reliable solution for critical care settings because it achieves precise environmental control, improves energy efficiency, and ensures compliance with strict healthcare facility standards.

## 1. Introduction

In recent years, intelligent buildings have developed at high speed worldwide, and Variable Air Volume (VAV) systems are widely used in air conditioning systems in most buildings. These systems are particularly well-suited for delicate settings like hospital operating rooms because they provide several advantages, such as increased thermal comfort, higher energy economy [1], and accurate environmental management. For patient safety as well as to reduce airborne contamination and preserve sterility during surgical procedures, it is crucial to maintain ideal air quality, temperature, humidity, and positive pressure in such areas [2].

VAV systems were first introduced in the mid-to late-1960s in response to Urban’s work [3]. Because of their capacity to modify the air supply in response to real-time zone-specific demands, they have become widely used in a variety of applications, such as residential buildings, healthcare facilities, industrial plants, and commercial complexes. The VAV is better in energy saving and maintaining indoor zone comfort conditions for supplying air temperature and flowrate based on the technology of variable frequency drives (VFD), which is integrated into Air Handling Units (AHUs), allow precise fan speed modulation based on analogue output control signals (e.g., 4–20 mA or 0–10 VDC) [4] issued by Direct Digital Controllers via the Building Automation System (BAS), where the BAS is a computer-based control architecture that facilitates centralized monitoring and management of various building systems, including heating, ventilation and air conditioning (HVAC), lighting, electrical, and low-voltage services. Typically, the BAS consists of control modules, instruments, and a personal computer (PC) equipped with the full Web-CTRL graphics software, server, and programming with a printer. The primary objective of the proper use of Building Automation Systems (BASs) is to enhance energy efficiency or cost-effectiveness while delivering superior performance. Additional primary roles of BASs are risk management, information, and facility management [5]. The VAV terminal units generally comprise four fundamental components: temperature and pressure sensors, a primary control module, a damper actuator, and a power supply unit [6].

This paper aims to maintain the required pressure and temperature inside the operating room (Surgical Suite) according to the mechanical design. As is known, the pressure in the operating room must be positive (i.e., it must be greater than the pressure in the external corridor), and the temperature, according to the ASHRAE Standard 170-2017 [2], ranges from 18 to 22 degrees Celsius (°C). This specified temperature is intended to ensure the flexibility of the doctor’s work and provide a suitable environment during the surgery, and also to prevent any effects that could damage medical devices when the temperature rises high; in addition to temperature, installing a humidifier within the AHU is necessary to ensure that the room air is safe and properly humidified (40% to 60% relative humidity) [2]. Regarding the pressure within the room, opening the operating room door leads to the entry of microbes and germs into the room; to prevent this, the air flow speed rate for supply fun of AHU must be increasing via supply VAV box in the operating room and positive pressure is achieved to expel germs and microbes, thereby preserving the patient’s health and minimizing their exposure to infection and the contaminated air extract by return fan of AHU from the operating room via return VAV box.

The proposed methodology is validated through real-world deployment at the Korean Iraqi critical care hospital project, located in the medical city complex in Baghdad, delivered to the Iraqi Ministry of Health. The Building Automation Systems (BASs) installed utilize Automated Logic Corporation (ALC) brands such as DDC panels with Web-CTRL software version 8.0 for full HVAC monitoring and control, including water tanks and service pumps, across the hospital infrastructure. For more information about this brand, the reader can refer to [7]. The system’s performance was evaluated across multiple operational scenarios to assess compliance with healthcare environmental standards, system robustness, and energy efficiency, confirming the effectiveness of the proposed approach.

To address the complex, nonlinear dynamics of operating room environments, this study proposes a cascade Proportional–Integral (PI) control strategy aimed at simultaneously regulating both temperature and differential pressure. The system integrates industry-grade components such as Automated Logic’s Web-CTRL software version 8.0, SE6166sp controllers (1150 Roberts Blvd. Kennesaw, GA 30144, USA), and a comprehensive array of field sensors and actuators to achieve coordinated, real-time control. An inner loop in the suggested control architecture dynamically modifies airflow to maintain positive pressure under a range of operating situations, while an outer loop regulates temperature depending on user-defined setpoints.

The contribution of this paper stands out from others by focusing on controlling two critical variables (temperature and pressure) simultaneously in two operating rooms with a single AHU. This is accomplished by designing and implementing a Building Automation System specifically for the healthcare field.

Each part of this paper is organized as follows, to enhance comprehension that will help you fully grasp this research: Section 2 goes into great length on the HVAC system in operating rooms, covering everything from the hardware and software to the thermal dynamics. In Section 3, we go into further detail about the control approach that was used, focusing on how the VAV system and the Building Automation System (BAS) components work together. Section 4 shows the experimental setup and explains how the performance testing will be performed in different real-world situations. In Section 5, we talk about empirical findings and performance analysis, which show that the system works and meets important healthcare requirements. Finally, Section 6 wraps up the work by going over the main points and suggesting possible areas for future research.

## 2. Operating Rooms HVAC System

### 2.1. Mechanical and Instrument Description

Multi-zone Variable Air Volume (VAV) systems are a key configuration in contemporary HVAC design, effectively providing thermal comfort in extensive and segmented environments, including commercial buildings, healthcare facilities, and industrial complexes [8]. The systems operate by distributing conditioned air from a centralized air handling unit (AHU) to various independently controlled zones. Terminal units, such as VAV boxes and motorized dampers, are utilized in each zone to adjust airflow dynamically in response to changing thermal loads, thus ensuring optimal environmental conditions specific to each zone.

**Figure 1:**
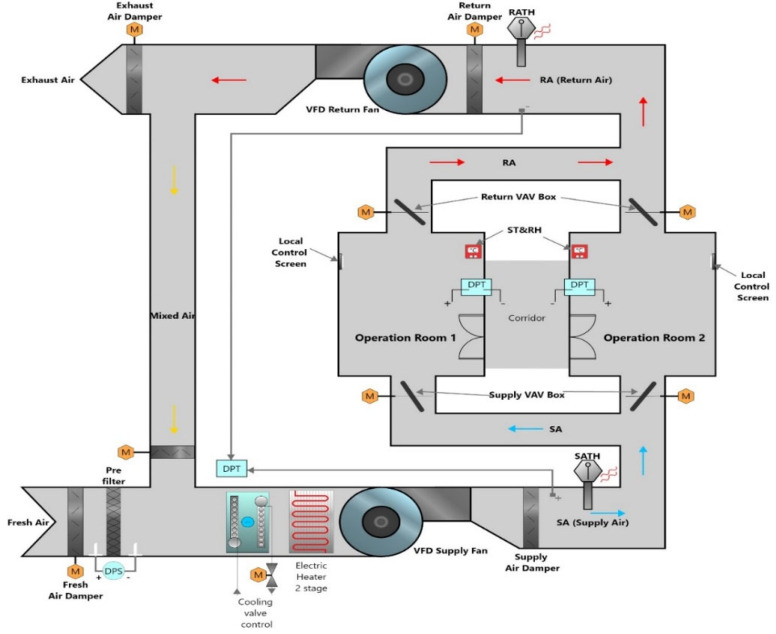
Schematic diagram for a multi-operating room of VAV system.

The current application, as depicted in Figure 1, demonstrates that the HVAC system for the operating rooms includes a complete set of mechanical and control components. The central AHU incorporates variable frequency drives (VFDs) for both supply and return fans, facilitating the modulation of fan speed in accordance with system demand. Air purification is accomplished via pre-filters and bag filters that remove particulate matter, including dust and pollen, before it reaches the cooling coil and then the supply fan. The air conditioning is subsequently directed through a chilled-water coil and a two-stage electric heater, followed by distribution through motorized dampers provided by Siemens [9] for fresh air intake, return, and exhaust. Each operating room is equipped with dedicated supply and return VAV boxes that include electronically actuated dampers for airflow control through voltage modulation (0–10 VDC). These motorized dampers are adjusted according to user-defined temperature setpoints from inside the room via a 7-inch local touch screen interface from Automated Logic [10]. This configuration enables surgical staff to achieve the desired system conditions for each operating room.

To ensure accurate control and monitoring, the system incorporates a suite of high-precision sensors and actuators:

- **Fan Speed Regulation** for supply and return lines is equipped with Danfoss VFDs [11]. According to ASHRAE (1999) [12], modulate the speed of airflow in response to readings from duct static pressure sensors and the aggregated opening and closing ratios of the VAV dampers in each room. The importance of this point is to maintain the static pressure setpoint and ensure adequate flow under design conditions.
- **Two-Way Modulating Valve** (0–10 VDC, Siemens) [13] controls the chilled water flow to the cooling coil, based on feedback from return air temperature sensors and control signals originating from the BAS workstation.
- **Differential Pressure Switch On/Off** (Sontay) [14] is configured experimentally with a threshold of 400 Pa to monitor filter cleanliness in case of any clogging by dust or contaminants; this will lead to exceeding the value and provide an alarm signal to the maintenance alarm.
- **A Differential Duct Static Pressure Transducer** (Carrier) [15] continuously measures supply duct pressure. As individual room temperatures approach their setpoints and their respective VAV dampers begin to close, the resultant pressure increase is detected and used to command a reduction in AHU fan speed, thus preventing over-pressurization, noise, or potential damage to the supply duct.
- **A Differential Pressure Transducer** (Sontay) [16] measures the pressure differential between the corridor (typically negative) and the operating room (positive). This input ensures that the system maintains the required positive pressure by proportionally adjusting both supply and return damper positions.
- **A Supply Air Temperature and Humidity (SATH) Sensor** (Sontay) [17] monitors the thermal and moisture content of the delivered air, ensuring that mechanical specifications are met during both heating and cooling modes.
- **A Return Air Temperature and Humidity (RATH) Sensor** (Sontay) [17], the feedback from the return air in both operating rooms is monitored within the common return duct, facilitating control over the chilled water flow into the cooling coil of the AHU through a two-way motorized valve.
- **Wall-mounted Room Temperature and Humidity (ST&RH) Sensor** (Sontay) [18] used to read the temperature and humidity of the operating room; based on these readings, the BAS systematically adjusts the opening of both supply and return Variable Air Volume (VAV) dampers to meet the designated temperature setpoint conditions.

The centralized DDC system effectively manages this integrated network of devices, offering a robust, responsive, and energy-efficient approach to meeting the strict environmental standards of surgical spaces.

### 2.2. Direct Digital Controller (DDC) Panel

**Figure 2:**
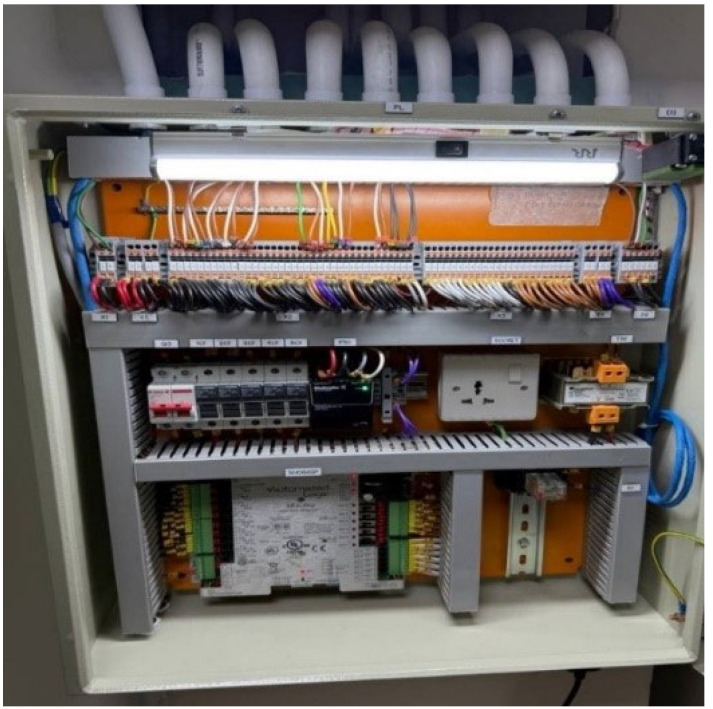
Layout of DDC panel.

**Figure 3:**
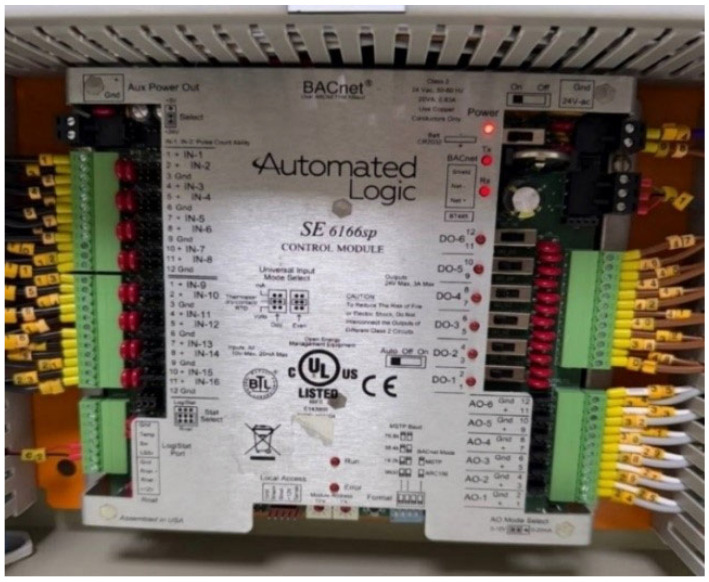
SE6166sp control module.

The SE6166sp single equipment controller module from Automated Logic is installed within the Direct Digital Control (DDC) panel to facilitate accurate regulation of temperature and pressure in hospital operating rooms. This module is installed with some of the electrical components, such as circuit breakers, relays, fuses, and terminal connections, as illustrated in Figure 2 and Figure 3. Engineered for high-performance control in single-equipment applications, the SE6166sp offers robust functionality and adaptability across diverse environments such as rooftop units, mechanical rooms, and air handling unit (AHU) systems.

The module features a comprehensive input/output configuration, including six digital outputs, six analogue outputs, and sixteen universal inputs. It supports BACnet communication over ARC156 or MS/TP protocols (ranging from 9600 bps to 76.8 kbps) for seamless integration with smart building control networks, so that the user can interact with the component for each function, such as heating, cooling, ventilation, and occupancy schedule [19]. Additionally, a Rnet port enables direct communication with ZS2 room sensors and equipment touchscreen interfaces developed by Automated Logic. Detailed technical specifications and hardware capabilities of this controller are documented in reference [20].

All system inputs and outputs, including start/stop commands, run/fault status indicators, environmental sensors, and motorized actuator signals, are precisely wired, labelled, and tested within the DDC panel during the commissioning phase prior to system activation. The DDC panel is connected via CAT6 LAN cable to the hospital’s main supervisory computer located in the central control room. Through this interface, operators can monitor system status in real time, respond to faults or alarms, and remotely adjust operational parameters for various HVAC subsystems, including chillers, cooling towers, and both primary and secondary pump sets.

### 2.3. Thermal Dynamics of the Operating Room

The thermal behavior of an operating room can be modelled using first-order differential equations derived from energy balance principles. The governing dynamic equation is the following [21]:

C_r_ × (dT_r_(t))/dt = ṁ_sa_ × c_p_ × (T_sa_(t) − T_r_(t)) + Q_int_(t) (1)

where

C_r_: Effective thermal capacitance of the room air [J/K].
T_r_(t): Room air temperature [K or °C].
ṁ_sa_: Supply air mass flow rate [kg/s].
C_p_: Specific heat of air [J/kg·K].
T_sa_(t): Supply air temperature [K or °C].
Q_int_(t): Internal sensible heat gain [W].

Assuming constant air properties and linearity, the system can be approximated as a first-order transfer function in the Laplace domain [21]:

(2) $$G(s)\;=\;(T_{r}(s))/(T_{\mathrm{sa}}(s))\;=\;K/(T_{s}\;+\;1)$$

*T*_*s*_ = C_r_/ṁ_sa_ C_p_ (3)

(4) $$G_{d}(s)=Tr(s)\;/{\;Q}_{int}(s)=1\;/\;(C_{r}\;*\;s+{ṁ}_{sa}\;*{\;C}_{p})$$

where

G(s): Transfer function from supply air temperature to room temperature.
K: Steady-state gain = 1.
$T_{s}$: Time constant [s].
$G_{d}(s)$: Transfer function from internal heat gains to room temperature [K/W].
$Q_{int}(s)$: Laplace transform of internal sensible heat gains [W].
s: Laplace variable [1/s].

This model serves as the plant for control system design and is commonly used for PI controller tuning.

### 2.4. Proportional Integral (PI) Controller

According to Haines [22], PI control is the optimal control approach for HVAC systems due to its enhancements in accuracy and energy efficiency relative to proportional control. A PI controller is a type of linear controller that calculates the control error based on the setpoint and process output, subsequently generating the controller output from the linear combination of the proportional and integral components of the error.

To regulate room temperature Tr(t) and maintain the required pressure differential, a Proportional–Integral (PI) controller is implemented. The PI controller’s output u(t), which drives the damper actuator or valve, is defined as follows [23]:

u(t) = Kp × e(t) + Ki × ∫ e(t) dt(5)

where

u(t): Control output.
e(t): Error signal (the difference between the setpoint and the measured process variable).
Kp: Proportional gain.
Ki: Integral gain.

In the Laplace domain, the controller transfer function becomes the following [23]:

C(s) = Kp + (Ki)/s(6)

The gains Kp and Ki can be tuned to achieve the desired transient and steady-state performance.

### 2.5. Web-CTRL Software Platform and Functional Components

Web-CTRL is a comprehensive, web-based building automation platform developed by Automated Logic. It provides facility managers and technical personnel with centralized, real-time access to monitor, control, and optimize various building systems. These include heating, cooling, ventilation, lighting, fire safety, security, backup power systems, and vertical transportation infrastructure such as elevators. Its remote accessibility enables efficient system oversight and rapid response from virtually any location. For detailed specifications, users may refer to [24].

**Figure 4:**
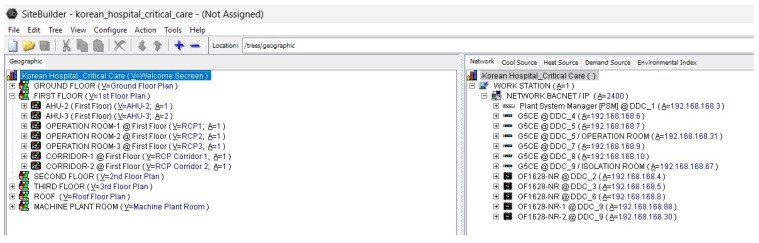
Web-CTRL Site Builder.

**Figure 5:**
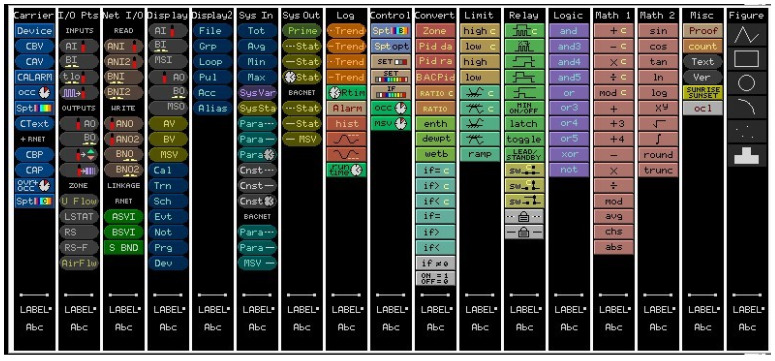
Web-CTRL Eikon Logic Builder micro-blocks.

**Figure 6:**
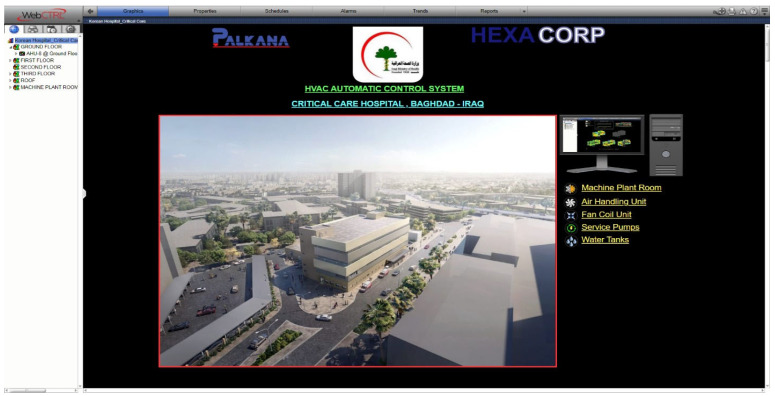
Web-CTRL View Builder.

The software environment is structured into several functional modules that support system integration, configuration, and visualization:

- Site Builder (illustrated in Figure 4): This module is used to define the overall building infrastructure, including floors, rooms, and the full inventory of mechanical and electrical systems to be managed through the BAS. It establishes the foundational architecture for project-specific configurations.
- Eikon Logic Builder (illustrated in Figure 5): Serving as the programming interface, this tool allows users to construct control logic through intuitive micro block programming. Engineers can implement system behavior based on operational requirements and design specifications, ensuring tailored automation logic for each application.
- View Builder (illustrated in Figure 6): This module facilitates the creation of graphical user interfaces (GUIs) that visually represent equipment status, control parameters, and real-time sensor data. The interactive dashboards simplify system navigation and allow operators to monitor and manage facility systems with ease and precision.

These components collectively create an integrated ecosystem that enhances operational efficiency, improves fault detection, and facilitates intelligent decision-making in complex building environments.

## 3. Operation and Control Strategy of Variable Air Volume Systems

VAV systems are engineered to adjust airflow to space dynamically by modulating the position of mechanical dampers. This modulation is accomplished via damper actuators incorporated in VAV terminal boxes, facilitating accurate regulation of conditioned air according to the thermal requirements of each zone. In situations where a room necessitates enhanced cooling, the associated VAV damper gradually opens to augment the supply of cold air until the target temperature is reached. If the space becomes overcooled, the VAV box damper actuator progressively closes to limit airflow [8].

The air pressure drop in the supply duct will happen when the VAV box damper actuator is opened based on the reading of the difference in duct static pressure by the DPT (differential pressure transducer) transducer and this indication will be sent to the DDC panels to alert the AHU’s supply fan to increase supply air delivery to the space. Conversely, if the space becomes very cool, the VAV box damper actuator is progressively closed to decrease the supply of cold air to the space. During the closing of the VAV box damper actuator, there is high pressure in the supply duct based on the reading of difference duct static pressure by DPT and this indication will be sent to the DDC panels to decrease the supply air delivery to space by the supply fan of AHU; this is usually applied in combination with variable-speed drives (VSDs) to supply the fan motor of AHU [12]. As a result, reduced airflow results in decreased fan power demand, leading to energy savings. This adaptive mechanism not only stabilizes system operation and satisfies the airflow under design conditions but also reduces fan energy consumption, contributing to overall energy efficiency [1]. The control strategies are essential to optimize the performance of VAV-based HVAC systems, particularly in complex or sensitive environments such as hospital operating rooms. Among the available techniques, Proportional–Integral–Derivative (PID) controllers are the most employed. These controllers combine proportional (P), integral (I), and derivative (D) actions to minimize control errors and improve system stability [4]. Depending on the control requirements, simplified variants such as PI and PD controllers may be used. The PI controller is particularly advantageous in HVAC systems, as it reduces rise time and eliminates steady-state error, while maintaining sufficient system responsiveness. On the other hand, PD controllers enhance damping characteristics and reduce overshoot, thereby improving transient performance and stability margins [25].

In the present work, a single AHU serves two separate operating rooms, as per the mechanical consultant’s specifications. To maintain independent control over temperature and pressure in each room, dedicated VAV boxes are installed on both the supply and return ducts. This configuration enables each space to preserve its unique environmental parameters regardless of the load conditions in adjacent rooms.

**Figure 7:**
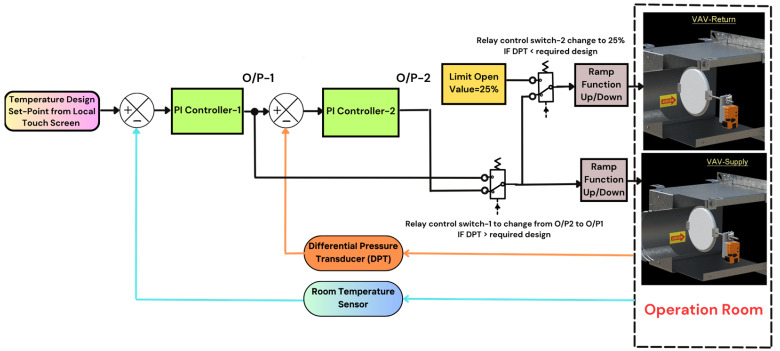
Cascade control system block diagram.

To meet the stringent thermal and pressurization requirements of surgical suites, a cascade PI control strategy is implemented. As shown in Figure 7, the system comprises two nested control loops. The outer loop governs temperature control and is driven by user-defined setpoints inputted via a wall-mounted touch screen interface [10]. This setpoint is continuously compared with real-time readings from a room temperature sensor [18]. The resulting error signal is processed by the first PI controller, which then generates a control output used as the reference signal for the inner loop.

The inner loop is responsible for pressure regulation. It compares the reference signal (output from the temperature loop) with feedback from a differential pressure transducer (DPT) [16] installed above the false ceiling of the room, which measures the pressure difference between the operating room (positive pressure) and the adjacent corridor (negative pressure). Maintaining a positive pressure differential is critical in surgical environments to prevent airborne contaminants from entering the sterile zone. In accordance with ASHRAE Standard 170-2017, operating rooms must sustain a minimum positive pressure of 2.5 Pa, with an ideal operational range of 7.5–12.5 Pa [2]; based on room volume and air exchange requirements, the project design specifies a pressure range of 3–15 Pa to ensure the positive pressure, and maintaining air quality and sterility is essential. So, the calculated Air Changes per Hour (ACH) is the number of times the air in a room is completely replaced with fresh air in one hour. It is a key factor in ventilation design, especially in critical spaces like operating rooms. The formula of ACH is the following [2]:

ACH = ((CFM × 60))/V(7)

where

ACH = Air changes per hour [1/h].
CFM = Supply airflow rate [ft^3^/min].
V = Room volume [ft^3^].
60 = Minutes in one hour (to convert CFM to hourly volume).

To convert the required airflow from m^3^/h to CFM (Cubic Feet per Minute), 1 m^3^/h = 0.589 CFM [2].

The cascade control approach ensures that temperature regulation is prioritized under normal conditions, while pressure control takes precedence in situations where sterility is at risk. This dual-loop configuration enables robust performance, enhanced stability, and compliance with healthcare environmental standards.

## 4. Performance Testing and Analysis

To evaluate the robustness and dynamic responsiveness of the proposed cascade PI control strategy, a series of performance tests was conducted under real operational conditions in a hospital environment. The primary objectives were to ensure the maintenance of a positive pressure differential within the operating rooms, as mandated by medical standards, and to sustain thermal comfort at a setpoint of 20 °C. Four distinct operating scenarios were analyzed for one of the two identical operating rooms; the same control logic and environmental behavior apply to the second room due to the system’s symmetrical configuration.

**Figure 8:**
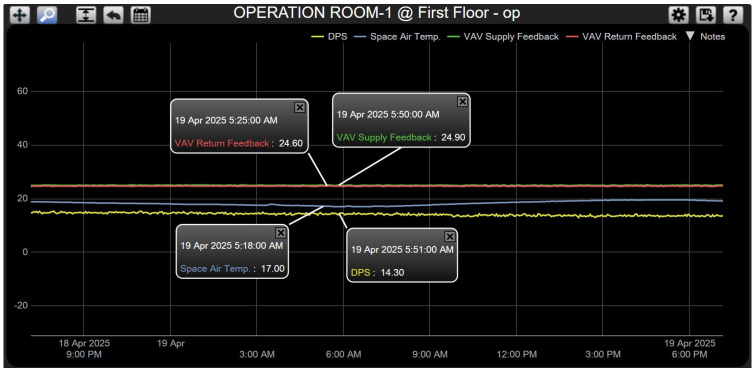
System response for Case 1.

**Case 1—Stable Conditions: No Control Action Required.** In this baseline scenario, the room temperature measured by the wall-mounted sensor was 17 °C, below the setpoint of 20 °C. Simultaneously, the pressure differential between the room and the adjacent corridor was within the acceptable range (14.30 Pa). Since both parameters already satisfied the design criteria, no corrective action was initiated by either of the two PI controllers. The motorized dampers of the supply and return VAV boxes were maintained at their minimum open position (around 25%), allowing continuous circulation of conditioned air to sustain occupant comfort. The system response for this steady-state condition is shown in Figure 8.

**Figure 9:**
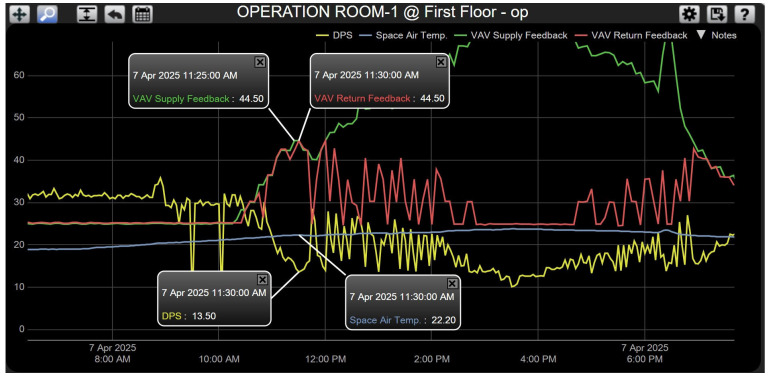
System response for Case 2.

**Case 2—Elevated Temperature with Normal Pressure.** In this dynamic scenario, the measured room temperature exceeded the setpoint; it was 22.20 °C above the setpoint of 20 °C, while the pressure differential remained within the desired range (13.50 Pa). As a result, the outer-loop temperature controller (PI-1) generated a corrective signal that served as a reference for the inner-loop pressure controller (PI-2). VAV dampers were modulated simultaneously using a unified control signal (an approximately 44.5% opening). This coordinated action allowed the system to gradually restore thermal equilibrium without compromising pressure stability. The controller continued adjusting damper positions until the room temperature converged to the setpoint. The performance trajectory of this control action is illustrated in Figure 9.

**Figure 10:**
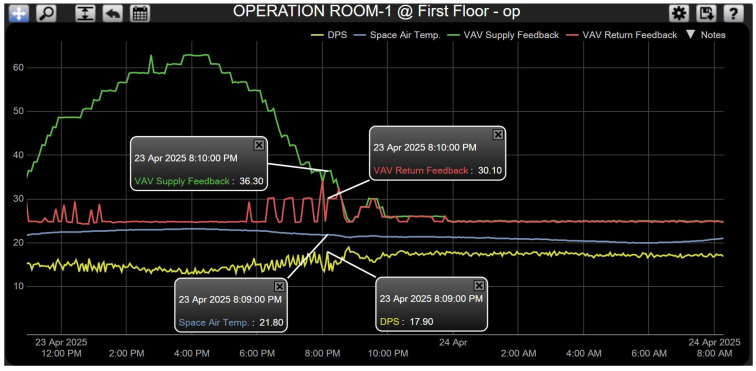
System response for Case 3.

**Case 3—Excessive Pressure: Prioritizing Thermal Control.** The pressure is greater than the normal range of 3 and 15 Pascals (17.9 Pascals); in this case, pressure is very high inside the room and satisfied, so PI controller-2 (output-2) is off by switching the relay control to PI controller-1 (output-1) (as shown in Figure 7) and this will be controlling and providing control signal (0–10 Vdc), depending on the first loop only for both motorized actuators of VAV boxes for the supply and return lines and based on the difference between room temperature setpoint (20 °C) and room/space temperature sensor, as shown in Figure 10 the VAV boxes for supply and return lines looking at room’s temperature (21.8 °C) being very close to room’s temperature setpoint (20 °C), so that both supply and return lines are closing in on one working percentage at the time where supply VAV box is opened at 36.3% and return is at 30.1% until both to be close each other with same percentage and this action will continue until the room’s temperature meets the room’s setpoint in this case it will revert back to Case 1. The system response for this scenario is shown in Figure 10. Normally, the high pressure happens when the supply and return VAV boxes of operating room-2 closing at minimum percentage of 25% and meet temperature and pressure room condition in this case all the cooling air will be going via the duct to operating room-1, so the pressure will be very high and once the operating room-1 satisfy and meet the room condition as shown and clarified in Case 1. In this scenario the differential duct static pressure (DPT) [15] in the supply line of the AHU will be send feedback signal to the BAS in order to reduce the motor speed by the VFD this lead to reduce the airflow in the supply duct and protect the motor itself and the duct from any damage because of high static pressure accumulated in the supply line [26].

**Figure 11:**
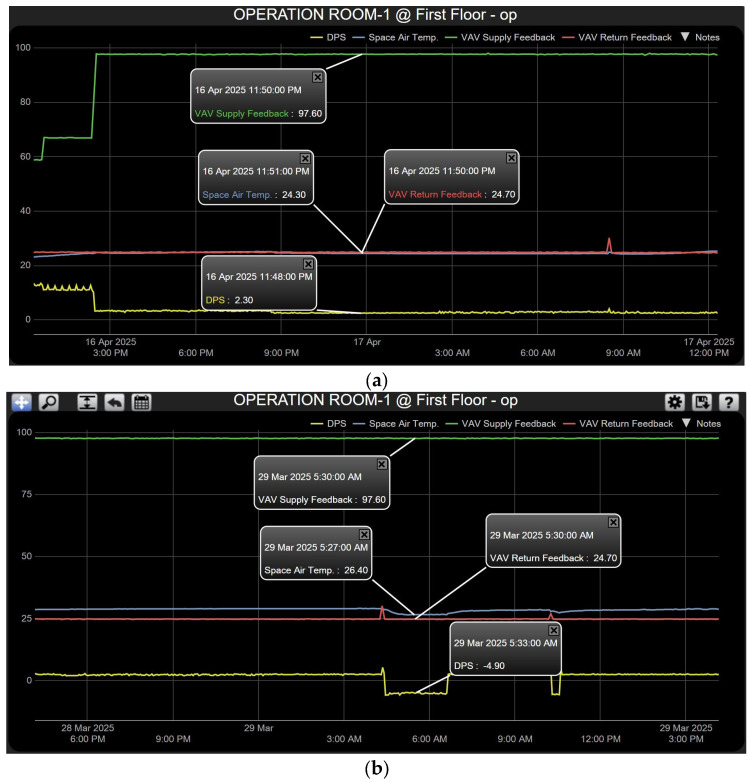
(**a**,**b**) System response for Case 4.

**Case 4—Pressure Deficiency: Overriding Temperature Control.** This critical case simulated a pressure drop below the minimum safe threshold. As shown in Figure 11a, the Differential Pressure Sensor between the corridor and operating room is 2.3 Pascals, which is less than the normal pressure range of 3–15 Pascals; therefore, the cascade control signal of output-2 will be directly modulating (0–10 Vdc) the motorized actuator of the VAV box for the supply line only. Since pressure is more critical and very low, to increase the pressure inside the operating room, the return VAV box must be opening less than the supply VAV box, so the relay control switch-2 change to limit open value is at 25% (as shown in Figure 7) and the supply VAV box is at the highest percentage at 100%; this action will continue to increase the airflow until the room’s pressure reaches the normal pressure range. Another situation, the loss of positive pressurization, in this case, as depicted in Figure 11b, the room’s pressure is in minus readings (−4.9 Pascals). In this case, the scenario will be the same and keep full open of the supply VAV box and the minimum limit open for the return VAV box until obtaining a positive pressure inside the operating room to prevent contamination from adjacent areas. Low pressure often happens when the AHU filter becomes dirty. This is detected by the Differential Pressure Sensor (DPS) [14], which sends a signal to the Building Automation System (BAS), and this will notify the hospital maintenance team to clean or replace the AHU’s filter, or the door of the operating room kept open for a long time. In this case, maintaining proper pressure takes higher priority than temperature control, as it has a critical impact on human life during surgery. During that time, the pressure will stabilize, and the temperature will gradually decrease to reach a comfortable room condition by cascade control action. 

Table 1 below gives a brief overview of the operational parameters for each scenario. It shows how the room temperature, pressure differences, and the damper opening percentages of the supply and return VAV boxes change when the control conditions change. This synthesis makes it easier to understand how systems react to different control and environmental challenges.

## 5. Discussion

The results show that the proposed cascade PI control strategy for VAV-based HVAC systems is effective and useful in real life, especially in hospital operating rooms and other critical healthcare settings. The use of two nested PI control loops, one for keeping the temperature stable and the other for keeping the pressure stable, makes it possible to control the environment reliably and adaptively in a wide range of operational situations. The hierarchical control logic of the proposed architecture is a major strength. The outer loop controls thermal comfort based on user-defined setpoints, while the inner loop makes sure that there is positive pressurization, which is necessary for infection control in surgical settings, as stated in ASHRAE Standard 170-2017. The dual-loop setup lets the system change its priorities on the fly. Under normal conditions (Case 1), it keeps the temperature comfortable without losing airflow efficiency. In some cases, like Case 4, the control strategy puts pressurization ahead of temperature accuracy to make sure the area stays sterile, even if it means that temperature accuracy is temporarily affected.

The test scenarios showed that the system could handle both gradual and sudden changes in the environment. By keeping damper movements from moving too much, the coordination of the two loops makes the system more stable and lessens wear on the actuators. High-resolution differential sensors, DDC panels, and Web-CTRL software work together to allow for real-time monitoring, diagnostic transparency, and operator intervention as needed.

Adding VFDs to the design of the AHU improves energy efficiency by allowing the fan speed to change based on pressure feedback (Case 3). The system does a good job of balancing the needs for comfort, safety, and energy efficiency on its own. Compared to traditional single-loop or threshold-based control systems, the cascade PI approach offers a stronger, more adaptable, and more context-aware solution that fits well with the needs of modern healthcare settings. The suggested method could be used as a model for future smart hospital infrastructure, especially in places where keeping the environment stable in real time is very important for patient safety and clinical outcomes.

## 6. Conclusions

This study built and thoroughly tested a cascade Proportional–Integral (PI) control system for a Variable Air Volume (VAV) system in a real hospital operating room. The dual-loop control architecture, which focuses on keeping the right pressure in the inner loop and the right temperature in the outer loop, does a great job of keeping the strict environmental conditions that healthcare standards require. Extensive real-world testing in four different operational cases showed that the system did an excellent job of keeping the target temperature range of 18–22 °C and a positive differential pressure of 3–15 Pascals. Specifically, in Case 1 (stable conditions), the operating room maintained 17.0 °C with +14.3 Pa, while in Case 2 (elevated temperature), the temperature rose to 22.2 °C with +13.5 Pa. In Case 3 (high pressure), the system achieved 21.8 °C with +17.9 Pa, and in Case 4 (low pressure), the pressure dropped to +2.3 Pa at 24.3 °C.

The results show that the proposed cascade PI control strategy works better than traditional control methods because it is more responsive, stable, and adaptable. The system quickly recovered from disturbances, like doors opening, which made sure that positive pressure was restored quickly, which is important for infection control. The smart changing of VAV box damper positions, along with Variable Frequency Drives (VFDs) for Supply Air Fans (SAFs) and Return Air Fans (RAFs), shows a lot of chances for improving energy efficiency while keeping important environmental factors the same. The combination of Automated Logic’s Web-CTRL and SE6166sp controllers in an industrial BAS shows how useful and scalable this solution is for modern healthcare facilities. This study shows a validated control solution that improves patient safety, optimizes HVAC system performance, and ensures that air quality and pressure standards are met in critical care hospital settings.

Future work could explore the incorporation of predictive control algorithms or adaptive PI tuning strategies to further enhance energy optimization and system resilience under varying operational demands by using another academic software, which is equipped with tools and intelligent control capabilities.

## Figures and Tables

**Table 1:** Comparative overview of temperature, pressure, and damper positioning in VAV system operational cases.

Cases	Operating Room Temperature (°C)	Operating Room Pressure (Pa)	VAV Supply % Open	VAV Return % Open
Case 1: Stable Conditions	17.0	14.3	24.9%	24.6%
Case 2: Elevated Temperature	22.2	13.5	44.5%	44.5%
Case 3: High Pressure	21.8	17.9	36.3%	30.1%
Case 4: Low Pressure	24.3	2.3	97.6%	24.7%
	26.4	−4.9	97.6%	24.7%

## Data Availability

All the data, information, and details collected from the site (Korean Iraqi critical care hospital) are included in the article; further inquiries can be referred to the corresponding author.

## Abbreviations

The following abbreviations are used in this manuscript:

PI	Proportional–Integral
VAV	Variable Air Volume
BAS	Building Automation System
ALC	Automated Logic Corporation
HVAC	Heating, Ventilation, and Air Conditioning
DDC	Direct Digital Controllers
VFDs	Variable Frequency Drives
AHUs	Air Handling Units
PC	Personal Computer
DPT	Differential Pressure Transducer
VSDs	Variable-Speed Drives
PID	Proportional–Integral–Derivative
ACH	Air Changes per Hour
CFM	Cubic Feet per Minute
DPS	Differential Pressure Sensor
